# ICT literacy, resilience and online learning self-efficacy between Chinese rural and urban primary school students

**DOI:** 10.3389/fpsyg.2022.1051803

**Published:** 2022-12-12

**Authors:** Jiaxin Li, Xinyi Huang, Xinyu Lei, Jiajie Wen, Minghui Lu

**Affiliations:** School of Education, Guangzhou University, Guangzhou, China

**Keywords:** primary school students, ICT literacy, online learning self-efficacy, student resilience, rural and urban areas

## Abstract

In the process of large-scale online education during the COVID-19 pandemic, students’ online learning has caused widespread public concerns. This study investigated the relationships between Chinese rural and urban primary school students’ information communications technology (ICT) literacy, student resilience, and online learning self-efficacy in a large-scale online education environment during the pandemic in China. We compared 5,037 primary school students in rural areas to 5,045 primary school students in urban areas with matching gender and grade in nine regions in China’s Guangdong province, using a survey comprising an ICT literacy scale, a student resilience scale, an online learning self-efficacy scale, and an ICT devices scale. The ICT literacy, resilience and online learning self-efficacy of primary school students in rural areas were significantly lower than those in urban areas (*p* < 0.01). The primary school students’ ICT literacy was significantly associated with student resilience and online learning self-efficacy. Student resilience played a mediating role between ICT literacy and online learning self-efficacy, while the rural/urban area factor moderated the relationship between ICT literacy and student resilience. These findings suggest that ICT literacy is more scarce and important for primary school students in rural areas of China than for those in urban areas. Improving ICT literacy among primary school students can enhance students’ resilience and thus improve their online learning self-efficacy, especially in rural areas.

## Introduction

At the end of 2019, the COVID-19 pandemic triggered a large-scale online teaching and learning boom in China. In the first half of 2020, China’s primary and secondary schools began the largest-scale online teaching in history to replace offline school education. According to statistics from China’s Ministry of Education (MOE), the official online cloud platform for primary and secondary schools in China received 2.073 billion page views and 1.711 billion visits in less than 3 months (MOE, 2020). At the same time, as online education often requires students to self-regulate their learning, and self-efficacy is an important indicator of the ability to self-regulate (Hodges, 2010), ensuring students’ online learning self-efficacy has become a topic of widespread concern. In the special circumstances of the COVID-19 pandemic, online learning has usually occurred at home through information and communications technology (ICT) devices (ICTDs). Therefore, differences in students’ ICT literacy (ICTL) are likely to lead to completely different learning experiences, influencing students’ psychological reactions to the learning process (Fraillon et al., 2019), as well as the sustainability and effectiveness of their learning (Hatlevik et al., 2018). For students who lack ICTL due to the difficulties and uncertainties they face in the learning process, the continuity and effectiveness of online learning is not guaranteed (Rohatgi et al., 2016). Worse still, such students are more likely to experience depression, burnout and anxiety (Lei et al., 2021). This implies that students’ psychological conditions during online learning may be largely related to their ICTL (Tran et al., 2020; Martin et al., 2021; Zhou and Yu, 2021).

It has been established that students’ ICTL may affect their resilience during online learning (Tran et al., 2020; Lei et al., 2021), and thus have an impact on their self-efficacy (Cheng et al., 2019; Grøtan et al., 2019). However, this mechanism has not been fully verified in large-scale online education processes. In addition, studies have also indicated that the relationship between students’ ICTL and resilience during online learning may vary among different groups (Tran et al., 2020). As China is considered to have a “digital divide” between urban and rural areas (Zhao et al., 2021), whether this difference exists between urban and rural areas in China remains to be verified.

Based on data on rural and urban primary school students in the specific context of large-scale online education in China during the pandemic, this study explored the relationship between students’ ICTL and online learning self-efficacy (OLSE) to verify the possible mediating effect of student resilience (SR) and provide suggestions for improving students’ OLSE. Meanwhile, considering the possible existence of a “digital divide” and differences in economic and social development between rural and urban areas in contemporary China (Zhao et al., 2021), this study collected the similar number of samples from rural and urban areas for comparative analysis while controlling for students’ gender and grade, and used the rural area/urban area factor as an important moderating variable to reveal the differences between ICTL and SR in rural and urban areas because the online education environment and resources differ between rural and urban China.

### Online learning self-efficacy

“OLSE” refers to students’ perceptions of their own abilities in the process of online learning (Artino and Mccoach, 2008). In education, self-efficacy has been shown to affect students’ choices of activities, effort invested, persistence, interests, and achievements, as well as their use of self-regulatory processes (Bandura, 1991; Schunk and Pajares, 2009). Researchers have argued that the self-directed nature of online learning makes self-efficacy a key component of academic success in distance education. For instance, students with higher online learning self-efficacy may be more interested in participating in online education, enjoy greater academic success (Martin et al., 2021), and experience more satisfaction and happiness during the learning process (Joo et al., 2013; Zhou and Yu, 2021). OLSE has been shown to be positively correlated with the quality of students’ experience of using ICT (Moos and Azevedo, 2009), proactive personality (Zheng et al., 2020), and interactions (learner–content interaction and learner–learner interaction) in the learning process (Wang et al., 2022).

### Information and communications technology literacy and OLSE

“ICTL” refers to an individual’s ability to effectively search for, organize, and process information from various digital media with a good understanding of technology systems and media forms (Ergul, 2004). Thus, technical processing skills, cognitive skills, and social values are combined in ICTL (Kereluik et al., 2013). Research has indicated that ICTL may help to protect children from the negative influence of the media, decrease information inequality, engage children fully in creative and social activities (Lee and Chae, 2012) and be positively related to students’ academic performance (Lei et al., 2021), especially in computer-based tests (Lau and Yuen, 2014).

In online education, ICTL may have a significant influence on self-efficacy. Researches showed that students with higher ICTL levels had more computer experience (Ranguelov et al., 2011), so they are more likely to complete online education tasks, have a positive learning experience, and thus have a greater sense of self-efficacy (International ICT Literacy Panel, 2012; Hatlevik et al., 2018). Research has also found that children who frequently use the Internet at home and school have higher levels of OLSE and ICTL than those who do not often use the Internet (Jackson et al., 2006). This means that ICTL may be positively correlated with OLSE (Fernández-Gutiérrez et al., 2020).

### Student resilience as a mediator of the relationship between ICTL and OLSE

SR is defined as students’ maintenance of positive adjustments during exposure to significant adversity (Luthar et al., 2010), which may reflect their psychological state in a specific learning situation. Researchers have commonly divided SR-related factors into two categories: individual personality attributes or dispositions, such as empathy, problem solving, and autonomy (Palma-Garcia and Hombrados-Mendieta, 2017); and environmental influences, such as family, school, and local community (Romano et al., 2021).

SR may mediate the relationship between ICTL and OLSE. To some extent, students’ ICTL predicts their SR in online learning (Tran et al., 2020), because SR reflects students’ psychological stability when they encounter online learning difficulties, which are often related to the use of ICT as an educational medium. For example, students who are not proficient in using instant messaging technology may find it difficult to interact with teachers (Lei et al., 2021). Higher ICTL means that students are more likely to solve problems on their own in the online learning process, thus ensuring their freedom of self-determination and creating a better learning experience; this helps increase the intrinsic motivation to engage in online learning (Ryan and Deci, 2000). Meanwhile, ICTL is considered to be positively related to students’ family background (i.e., socio-economic status and parents’ educational level), while a better family environment helps students to face online risks more positively, thus improving SR (Tran et al., 2020). Higher ICTL can help students to develop resilient responses by using technology as a mechanism to deal with adverse situations, and create opportunities for social support and positive relationships in online learning, thus improving their learning experience and SR (Rosenberg et al., 2018).

SR may also be related to OLSE in online learning. Psychological research has found that self-efficacy is often positively correlated with personal resilience and perseverance (Bandura, 1986). Those who think more positively about situations and feel less depressed and powerless tend to have a higher sense of self-efficacy (Bandura, 1997). Researchers have pointed out that SR can positively predict learning self-efficacy (Cheng et al., 2019). Learners with high resilience usually face pressure or challenges with positive attitudes and actions to promote self-development and self-efficacy (Kamtsios and Karagiannopoulou, 2013), while lower SR often means running the risk of experiencing diminished academic self-efficiency (Grøtan et al., 2019). In the network-based learning environment, SR is also considered to positively predict learners’ self-commitment and OLSE (Kuo et al., 2013). Some empirical studies on online education during COVID-19 have indicated that SR may have a significant predictive effect on student learning outcomes and OLSE (Martin et al., 2021; Warshawski, 2022).

### The moderating effect of the rural/urban area factor between ICTL and SR

Students’ ICTL may influence SR differently in different environments (Tran et al., 2020). Because the marginal effect of scarce resources is always stronger than that of non-scarce resources (Zhang et al., 2019), the influence of students’ ICTL on SR may be greater in underdeveloped regions than in developed regions. For instance, research has found that the influence of students’ motivation to use ICT on their actual ICT use behavior varies significantly between regions (Maldonado et al., 2009). In China, the “digital divide” between RAs and UAs has long been acknowledged (Zhao et al., 2021). In China, RAs are complex areas with natural, social, and economic characteristics. They serve multiple functions such as agricultural production, residence and ecology, and include towns and villages outside the urban areas. RAs are usually characterized by a sparser population and weaker economies due to poor development or unavailability of the required infrastructure. In contrast, UAs are residential areas where the non-agricultural population is concentrated and engaged in non-agricultural production activities such as industry and commerce (Lu and Chen, 2004). As the center of social, economic, and cultural activities within a certain geographical area, UAs often have huge advantages over RAs in terms of ICT infrastructure (Kumar and Kumara, 2018). Students in UAs also have much more access to ICTDs and ICTL than those in RAs do (CNNIC, 2021). Research has shown that ICTL may have a stronger relationship with willingness to use technology and resilience in using technology in online education for Chinese RA participants compared with Chinese UA participants (Martin et al., 2021). In view of the “digital divide” between UAs and RAs in China, rural primary school students may face more scarce resources and uncertainties such as a worse Internet connection in online learning (Wu et al., 2019); thus, the influence of ICTL on SR may be more obvious and profound for students in RAs.

### The study

**Figure 1:**
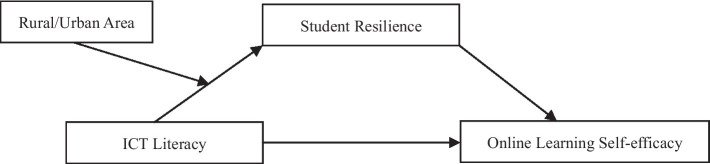
Research model.

Existing studies have revealed the relationship among ICTL, SR and OLSE to some extent, but it has not been fully verified in large-scale online education environments. Meanwhile, most studies have focused more on college and secondary school students than on elementary school students, due to their relatively high participation in online education in general (Kuo et al., 2013; Grøtan et al., 2019; Lei et al., 2021). The purpose of this study was to explore the relationship between Chinese students’ ICTL and OLSE in a large-scale online education environment during the COVID-19 pandemic, and to verify the mediating role of SR in this relationship and the moderating effect of the rural/urban area between ICTL and SR. The following hypotheses were verified using a moderated mediation model (Figure 1):

*Hypothesis 1*: ICTL is positively related to OLSE (Martin et al., 2021; Wang et al., 2022).

*Hypothesis 2*: SR mediates the relationship between ICTL and OLSE (Rosenberg et al., 2018; Cheng et al., 2019).

*Hypothesis 3*: The RA/UA factor moderated the relationship between ICTL and SR, and the positive correlation between ICTL and SR may be stronger in RAs than in UAs (Wu et al., 2019).

## Materials and methods

### Procedure

In this study, a cross-sectional questionnaire survey was conducted to investigate the online learning situation of primary school students in China’s Guangdong province during the COVID-19 pandemic. To ensure that the sample was reasonably distributed across regions and reduce the shadow of common method deviation (CMV) caused by the measurement methods (Podsakoff et al., 2003), the study adopted a combination of objective sampling and stratified random sampling, invited relevant education authorities to assist with the distribution and recovery of questionnaires by directional distribution and recovery, and informed the participants of the expected significance of the study, promising that the questionnaire data would be completely confidential. The families of 10,381 primary school students who participated in online learning during the pandemic were investigated. Because primary school students may find it difficult to understand questions on this topic or objectively state their own experiences, parents of the students were asked to assist their children in answering the questionnaires. To avoid possible bias caused by students and parents answering the questions together, researchers repeatedly stressed the importance of data, and asked the participants to answer objectively (Podsakoff et al., 2003). The instrument for the measurement was the web-based questionnaire platform “Sojump,” and participants were requested to submit all data through this platform. All of the participants took part in the survey voluntarily and did not receive any monetary compensation. As the study involved human participants, it was reviewed and approved by the ethics committee of the authors’ institution. This study was a cross-sectional study, and the data were analyzed using a moderated mediation model.

### Participants

All of the 10,100 invited participants completed and returned the questionnaire survey. If the questionnaire responses showed that the primary school students in the families surveyed had not participated in any form of online learning during the pandemic, these questionnaires were excluded. Manual screening showed that 10,082 (99.82%) questionnaires were valid, including 5,037 from rural areas and 5,045 from urban areas. The chi-square tests showed that there were no significant differences in gender and grade between rural and urban samples (χ^2^ = 0.00, *p* > 0.05).

### Measures

#### Social demographic survey

The questionnaire collected some information about the primary school students and their parents (such as each students’ gender and grade, family location and income, and parents’ education level). Samples in RAs and UAs had achieved quantitative matching in students’ gender and grade (*χ*^2^ = 0.00, *p* > 0.05). In the samples of RAs, 3,987 mothers (79.15%) and 1,050 fathers (20.85%) were included, and in the samples of UAs, 4,013 mothers (79.54%) and 1,032 fathers (20.46%) were included. For more details, please refer to Table 1.

**Table 1:** Demographic characteristics of the participating guardians and students for RAs and UAs.

	RAs (*n* = 5,037)	UAs (*n* = 5,045)	*χ* ^2^
**Student’s gender**			
Female	2,518 (49.99%)	2,524 (50.03%)	0.00
Male	2,519 (50.01%)	2,521 (49.97%)	
**Participant**			
Mother	3,987 (79.15%)	4,013 (79.54%)	0.23
Father	1,050 (20.85%)	1,032 (20.46%)	
**Mother’s educational level**			
Primary education and below	333 (6.61%)	165 (3.27%)	710.62***
Secondary education	3,245 (64.42%)	2,103 (41.68%)	
Tertiary education and above	1,459 (28.97%)	2,777 (55.05%)	
**Father’s educational level**			
Primary education and below	245 (4.86%)	102 (2.02%)	964.53***
Secondary education	3,414 (67.78%)	2026 (40.16%)	
Tertiary education and above	1,378 (27.36%)	2,917 (57.82%)	
**Annual household income (in RMB)**			
≤50 K	1,410 (27.99%)	1,223 (24.24%)	94.85***
50–90 K	576 (11.44%)	427 (8.46%)	
90–120 K	1,049 (20.83%)	917 (18.18%)	
≥120 K	2002 (39.74%)	2,478 (49.12%)	

*p < 0.05, **p < 0.01, ***p < 0.001. USD1 ≈ 6.38RMB.

#### Student ICTL scale

ICTL was measured using the student ICTL scale from the 2018 International Computer and Information Literacy Study of the International Association for the Evaluation of Educational Achievement (Fraillon et al., 2019), abridged based on the actual situation in China. The abridged scale contained seven items measuring the students’ ability to use ICT for learning, such as whether they could use ICT to “search for and find relevant information for a school project on the Internet.” Each item was scored as 0 (“I do not think she/he could do this”), 1 (“she/he has never done this but he/she could work out how to do this”), or 2 (“she/he knows how to do this”), with higher scores representing higher levels of student ICTL. This scale was a unidimensional scale and had been shown to have good reliability and validity in international surveys (Fraillon et al., 2019). Cronbach’s α for this scale was 0.82.

#### Family possession of ICTDs scale

A family possession of ICTDs scale was derived from a 2019 report on children’s media literacy by the United Kingdom Office of Communications (OFCOM, 2020) to investigate families’ ownership of ICTDs. The scale was abridged based on the situation in China. The abridged scale measured whether the students’ families owned 10 kinds of ICTDs, such as desktop or laptop computers. Each question was scored from 0 to 3. The higher the score, the more types of ICTDs the family owned. This scale was a unidimensional scale and had been shown to have good reliability and validity in large-scale surveys (OFCOM, 2020). Cronbach’s α for this scale was 0.65.

#### SR scale

SR was measured using the SR scale developed by Lereya et al. (2016), abridged based on the actual situation in China. The abridged scale consisted of 10 items focusing on students’ mental health and sustainability of learning activities, such as whether the students “believes that he/she can do most things if he/she tries” when studying online during the pandemic. Each item was scored as 0 (“no”) or 1 (“yes”). The higher the total score, the stronger the SR. This scale was a unidimensional scale and had been shown to have good reliability and validity in research (Lereya et al., 2016). Cronbach’s α for this scale was 0.78.

#### OLSE scale

In this study, formative assessment was used to measure students’ OLSE, which to some extent reflects their satisfaction and efficiency as online learners (Blayney and Freeman, 2008). The scale was excerpted from the Students’ Evaluation of Educational Quality (Marsh and Roche, 1997), and supplemented with some other questions in the questionnaires reflecting students’ OLSE (Bidder et al., 2016; Tian et al., 2017), in order to better match the actual situation of large-scale online education in China. The scale consisted of 10 questions, such as whether students “have clear and appropriate learning goals” in the process of online learning. The score for each item ranged from 0 (“very inaccurate”) to 4 (“very accurate”). The higher the score, the better the student’s OLSE. This scale was a unidimensional scale and had been shown to have good reliability and validity in research (Marsh and Roche, 1997). Cronbach’s α for this scale was 0.93.

### Statistics

SPSS 26.0 was used to generate the mean and standard deviation of each variable, and Pearson correlation analysis was used to explore the relationships between the variables. The PROCESS 3.5 macro for SPSS was used to test the mediating and moderating effects of the hypotheses (Hayes, 2018), and bootstrapping (5,000 bootstrap samples) was used to generate the 95% confidence intervals (Cis). If there was no 0 in the CI or the value of p was less than 0.05, the coefficient was considered statistically significant. The model took the total ICTL score (range 0–14) as the independent variable, the total SR score (range 0–10) as mediating variable, the total OLSE score as the dependent variable (range 0–40), RA/UA as the moderating variable (dummy variable), and the total ICTD score (range 0–30) as the control variable. All of the variables except for RA/UA were standardized before estimation, which made one standard deviation the metric of difference on the variables (Frazier et al., 2004).

## Results

The results of this study were presented from the following three aspects. Firstly, the CMV test and collinearity diagnostic test were used in subsection “Common method variance test and collinearity diagnostic test” to determine the reliability of the data and the model. Secondly, in subsection “Descriptive statistics, correlations and differences between RAs and UAs”, descriptive statistics and difference tests were used to reveal the main characteristics of Chinese primary school students in ICTL, SR and OLSE during the online learning process, as well as the differences between rural and urban primary school students in each variable. It could be important to understand the overall situation of large-scale online education in China. Finally, in order to test hypotheses 1, 2 and 3, the relationship between students’ ICTL and OLSE, as well as the mediating role of SR and the moderating role of the RA/UA factor were analyzed in subsection “Verification of hypotheses 1, 2 and 3: a moderated mediation model test.”

### Common method variance test and collinearity diagnostic test

Hausman’s single-factor test was used to test the common method variance (CMV). The results showed that the initial eigenvalues of seven factors were over 1, and the variance of the first factor was interpreted as 22.20% (lower than the critical value of 40%), indicating that there was no serious CMV problem (Podsakoff et al., 2003). The variance inflation factor (VIF) was used to test for multicollinearity among variables. The results showed that all values <10, average >1, and tolerance >0.1, indicating that there was no multicollinearity problem (Bowerman and O'Connell, 1992). Therefore, the data were reliable.

### Descriptive statistics, correlations and differences between RAs and UAs

The questions and answers (mean scores & standard deviations) of each scale for RAs and UAs, and the results of t-tests and chi-square tests between the two areas are shown in Tables 2–4. The results showed that the ICTL, SR and OLSE on all questions in the samples of RAs were significantly lower than those in the samples of UAs (*p* < 0.01). This means that there may be a “digital divide” between RAs and UAs, and rural primary school students are weaker than urban primary school students in ICTL, SR, and OLSE. Moreover, according to the descriptive statistics, large-scale online education during the pandemic showed obvious “teacher-centered” characteristics, and the students’ initiative to participate in educational activities was insufficient. For instance, for ICTL, the mean scores for “use spreadsheet programs” (0.24/0.29), “collaborate with others by sharing resources” (0.27/0.32), and “make any type of presentations” (0.29/0.38) were significantly low. In OLSE, the mean scores for “have clear and appropriate learning goals” (1.33/1.48), “make full use of various learning resources and arrange learning” (1.38/1.52), and “actively discuss with teacher and classmates on the Internet” (1.54/1.76) were also obviously low. These low scores indicate that these areas require the attention of educators in China.

**Table 2:** Questions and mean scores and standard deviations of the ICTL scale for the samples of RAs and UAs.

	RAs (*M* ± *SD*)	UAs (*M* ± *SD*)	*t*
1. Find useful learning resources on the Internet	1.24 ± 0.82	1.30 ± 0.80	−4.21***
2. Participate in discussions on Internet platforms (e.g., Weibo, WeChat, QQ)	0.79 ± 0.84	0.87 ± 0.84	−4.34***
3. Make any type of presentations (e.g., Microsoft PowerPoint, WPS and other programs)	0.29 ± 0.61	0.38 ± 0.69	−7.05***
4. Use the Internet for online purchase and payment	0.42 ± 0.69	0.50 ± 0.73	−5.79***
5. Use spreadsheet programs (e.g., Microsoft Excel) to save or analyze data	0.24 ± 0.57	0.29 ± 0.62	−4.72***
6. Collaborate with others by sharing resources (e.g., QQ documents, Baidu cloud)	0.27 ± 0.59	0.32 ± 0.63	−4.75***
7. Use online learning management system (e.g., Tencent classroom, rain classroom, enterprise WeChat)	0.66 ± 0.84	0.76 ± 0.87	−6.37***

0, I do not think she/he could do this; 1, She/He has never done this but he/she could work out how to do this; 2, She/He knows how to do this. *p < 0.05, **p < 0.01, ***p < 0.001.

**Table 3:** Questions and answers of the SR scale for the samples of RAs and UAs.

	RAs (YES/NO)	UAs (YES/NO)	*χ* ^2^
1. Do things at home that make a difference (i.e., make things better)	90.07%/9.93%	91.66%/8.34%	7.60**
2. Help her/his family make decisions	73.85%/26.15%	80.20%/19.80%	57.34***
3. Can work out her/his problems	68.85%/31.15%	75.14%/24.86%	49.52***
4. Can do most things if she/he try	84.67%/15.33%	87.27%/12.73%	14.14***
5. There are many things that she/he do well	78.48%/21.52%	82.12%/17.88%	21.13***
6. Feel bad when other people get their feelings hurt	85.82%/14.18%	87.67%/12.33%	7.47**
7. Try to understand what other people feel	83.20%/16.80%	85.13%/14.87%	7.04**
8. Know where to go for help when she/he has a problem	90.33%/9.67%	92.71%/7.29%	18.30***
9. Have goals and plans for future	67.66%/32.34%	70.60%/29.40%	10.25**
10. Think she/he will be successful when she/he grows up	76.26%/23.74%	78.91%/21.09%	10.21**

*p < 0.05, **p < 0.01, ***p < 0.001.

**Table 4:** Questions and mean scores and standard deviations of the OLSE scale for the samples of RAs and UAs.

	RAs (*M* ± *SD*)	UAs (*M* ± *SD*)	*t*
1. Have clear and appropriate learning goals	1.33 ± 0.79	1.48 ± 0.88	−8.86***
2. Understand the learning goals set by teacher	1.61 ± 0.84	1.76 ± 0.95	−8.51***
3. Make full use of various learning resources and arrange learning	1.38 ± 0.86	1.52 ± 0.93	−7.64***
4. Take online classes on time	1.80 ± 1.02	2.00 ± 1.09	−9.64***
5. Keep up with teacher’s progress and complete the learning content	1.82 ± 0.91	2.01 ± 1.01	−10.04***
6. Preview and review in time	1.62 ± 0.89	1.79 ± 0.97	−8.84***
7. Complete homework on time and earnestly	1.99 ± 0.96	2.13 ± 1.02	−7.41***
8. Satisfied with the learning process and academic performance	1.56 ± 0.92	1.76 ± 1.01	−10.28***
9. Actively discuss with teacher and classmates on the Internet	1.54 ± 0.87	1.76 ± 0.96	−11.88***
10. Satisfied with the quantity and quality of knowledge acquired	1.55 ± 0.86	1.75 ± 0.96	−11.20***

0, very inaccurate; 1, basically inaccurate; 2, moderately; 3, basically accurate; 4, very accurate. *p <0.05, **p < 0.01, ***p < 0.001.

The mean score, standard deviation and correlation on all variables are shown in Table 5. The mean total scores of ICTD, ICTL, SR and OLSE were 11.29 (SD 4.75), 4.17 (SD 3.55), 8.15 (SD 2.21) and 17.08 (SD 7.44). There was a significant correlation between every two variables, and the correlation coefficients between OLSE and ICTL, OLSE and SR, SR and ICTL were 0.349 (*p* < 0.001), 0.325 (*p* < 0.001), and 0.259 (*p* < 0.001).

**Table 5:** Descriptive statistics and correlations.

	ICTD(C)	ICTL(X)	SR(M)	RA/UA(W)	OLSE(Y)
ICTD(C)	-				
ICTL(X)	0.367***	-			
SR(M)	0.216***	0.259***	-		
RA/UA(W)	0.211***	0.075***	0.073***	-	
OLSE(Y)	0.292***	0.349***	0.325***	0.118***	-
M ± SD	11.29 ± 4.75	4.17 ± 3.55	8.15 ± 2.21	-	17.08 ± 7.44

*p < 0.05, **p < 0.01, ***p < 0.001.

### Verification of hypotheses 1, 2 and 3: A moderated mediation model test

**Figure 2:**
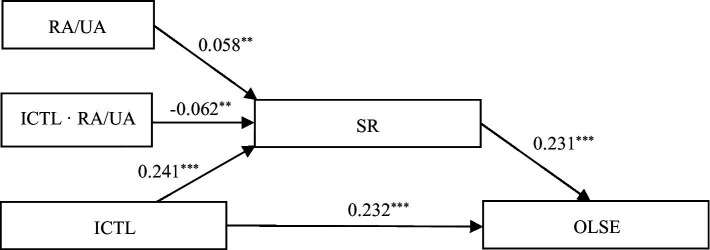
Mediating and moderating effects.

To test the validity of hypotheses 1, 2, and 3, the standardized variables (Z scores) were put into the PROCESS 3.5 macro to test the moderated mediation effect after controlling for the ICTDs owned by the students’ families. As shown in Table 6, SR was identified as an important intermediary between ICTL and OLSE. ICTL significantly positively predicted SR (a_1_ = 0.241, CI = [0.213, 0.269]) and SR significantly positively predicted OLSE (b = 0.231, CI = [0.212, 0.249]). When SR was added, ICTL still significantly positively predicted OLSE (c’ = 0.232, CI = [0.213, 0.251]). The confidence interval did not include 0, indicating that SR played a partial mediating role between ICTL and OLSE (Figure 2). The results showed a significant positive correlation between the primary school students’ ICTL and OLSE during online learning, and SR played a mediating effect, supporting hypotheses 1 and 2.

**Table 6:** Moderated mediation analysis (*n* = 10,082).

	SR					OLSE				
		*B*	*SE*	*t*	*CI*		*B*	*SE*	*t*	*CI*
ICTL	a_1_	0.241	0.015	16.632***	(0.213, 0.269)	c’	0.232	0.010	23.759***	(0.213, 0.251)
SR		-	-	-	-	b	0.231	0.009	24.804***	(0.212, 0.249)
RA/UA	a_2_	0.058	0.020	2.964**	(0.020, 0.096)		-	-	-	-
ICTL · RA/UA	a_3_	−0.062	0.019	−3.227**	(−0.099, −0.024)		-	-	-	-
ICTD	f	0.133	0.010	12.711***	(0.112, 0.153)	g	0.157	0.010	16.297***	(0.138, 0.176)
		*R*^2^ = 0.086 *F*(4, 10,077) = 235.914***					*R*^2^ = 0.202 *F*(3, 10,078) = 849.250***			

*p < 0.05, **p < 0.01, ***p < 0.001.

The results of the moderating effect test showed a significant interaction between the RA/UA factor and the primary school students’ ICTL (a_3_ = −0.062, CI = [−0.099, −0.024]), indicating that the moderating effect was statistically significant. To test the indirect effect of ICTL on OLSE under the moderating effect, a bootstrap test (samples = 5,000) of ICTL effect of RA and UA primary school students was conducted. As shown in Table 7, the mediation effect was significant for both RAs and UAs (CI_RA_ = [0.048, 0.063], CI_UA_ = [0.035, 0.048]) and the moderated mediation effect index a_3_ · b with regulation was significant (a_3_ · b = −0.014, CI = [−0.022, −0.006]). The test results showed a moderated mediation effect, verifying hypothesis 3.

**Table 7:** Conditional indirect effects of X on Y at values of the moderator and index of moderated mediation.

	Indirect effect/index	Boot SE	Boot LLCI	Boot ULCI
RA	0.056	0.004	0.048	0.063
UA	0.041	0.003	0.035	0.048
a_3_ · b	−0.014	0.004	−0.022	−0.006

**Figure 3:**
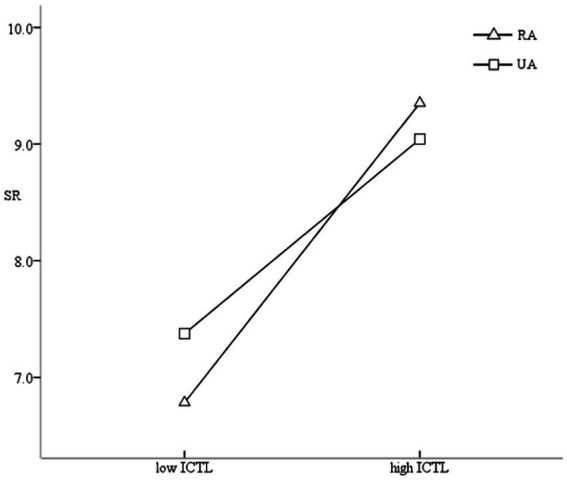
Moderating effect of RA/UA (unstandardized scores).

A simple slope was used to analyze the moderating effect of RA/UA between ICTL and SR. The results showed that the ICTL of the RA primary school students had a significant positive predictive effect on their SR (*β*_RA_ = 0.290, CI_RA_ = [0.261, 0.319]). The ICTL of the UA primary school students also significantly positively predicted their SR, but the predictive effect was smaller than that for the RA students (*β*_UA_ = 0.224, CI_UA_ = [0.199, 0.249]). This showed that for the RA primary school students, compared with their UA counterparts, ICTL played a more important role in predicting their SR (Figure 3).

## Discussion

The findings of this study were discussed from the following three aspects. Firstly, the ICTL, OLSE, and SR characteristics of Chinese primary school students were discussed in the first subsection, which may help educators to better understand the overall situation of students during the large-scale online education in China, and provide inspiration for them to improve online teaching. Secondly, in the second subsection, the relationship between ICTL and OLSE, and the mediating role of SR were discussed. This may be helpful to understand the importance of enhancing students’ ICTL and maintaining their mental health and emotional stability for online education. Finally, the moderating effect of RA/UA factor were discussed in the third subsection, which may help to arouse policy makers’ attention to improving ICTL for rural students in China, so as to bridge the gap between urban and rural students in online learning.

### ICTL, OLSE, and SR characteristics of Chinese primary school students

The overall data (Tables 2–4) indicated that in the process of large-scale online education during the pandemic in China, the sampled primary school students’ ICTL scores were generally not high, especially for “make presentations,” “use spreadsheet programs to save or analyze data,” and “collaborate with others by sharing resources.” This showed that the students may have mostly used ICTDs for information retrieval, rather than interacting with others (Yang, 2017). In terms of SR, most of the students thought that they were resilient during online learning, especially for the items “know where to go for help” and “do things at home that make a difference.” However, the scores for “have goals and plans for the future” and “can work out her/his problems” were relatively low, indicating that the students’ online learning may have depended on the guidance of their parents and teachers to an extent (Deng et al., 2020). Similarly, in the OLSE scale, the scores for indicators reflecting the students’ compliance with their teachers’ instructions were often high, such as “complete homework on time and earnestly” and “keep up with teacher’s progress,” while the scores for indicators reflecting the students’ learning ability and initiative were relatively low, such as “have clear and appropriate learning goals” and “make full use of various learning resources and arrange learning.” This showed that the online education process was dominated by teachers and that the students’ active behavior was limited, which may have been related to China’s long-standing educational tradition characterized by teacher-centered didactic pedagogies (Deng et al., 2020).

### The influence of ICTL on OLSE, and the mediating role of SR (hypotheses 1 and 2) were verified

The results of this study showed that the primary school students’ ICTL significantly influenced their SR and OLSE when controlling for their families’ ownership of ICTDs. This indicates that in the context of large-scale online education during the COVID-19 pandemic, ICTL is an important factor affecting primary school students’ online learning experience and self-efficacy, as well as their emotional stability during the learning process (Lei et al., 2021). This finding demonstrates the importance of improving students’ ICTL and bridging gaps in ICTL between different groups (Wu et al., 2019), because the ICTL differences among students may further aggravate the differences in SR and OLSE among them (Hatlevik et al., 2018; Tran et al., 2020). ICT education should be strengthened to continuously improve primary school students’ ICTL in an era of large-scale online education caused by crises such as the COVID-19 pandemic (Lei et al., 2021).

SR partially mediated the relationship between primary school students’ ICTL and OLSE. Higher ICTL can improve students’ participation in online learning and their ability to deal with related tasks and difficulties, thus improving their mental health and emotional stability, or SR (Tran et al., 2020). Greater SR means that students have a more comfortable online learning experience and are more likely to gain a sense of accomplishment from studying, which promotes their OLSE (Martin et al., 2021). This finding demonstrates the importance of psychological factors in the context of large-scale online education. Improving SR in the learning process may be an important way to improve students’ OLSE (Cheng et al., 2019). In the process of large-scale online education, therefore, teachers and parents need to continuously pay attention to primary school students’ psychological and emotional state (Warshawski, 2022). It is necessary to adjust the content and design of online education according to the prevailing situation (Zheng et al., 2020) or to provide psychological interventions in time (Romano et al., 2021) to ensure students’ mental health and improve SR.

### The moderating effect of the RA/UA factor in the relationship between ICTL and SR (hypothesis 3) was verified

The sample of rural primary school students was significantly weaker than the urban primary school sample in ICTL, SR and OLSE. At the same time, compared with the sample of urban primary school students, the rural primary school sample showed a stronger positive correlation between ICTL and SR. For rural students, the quality of online learning experiences may be more closely related to ICTL (Martin et al., 2021); therefore, improving their ICTL may be more helpful in improving their resilience and psychological stability in the learning process. The moderating effect of the RA/UA factor may be caused by the long-standing “digital divide” between UAs and RAs in China (CNNIC, 2021). Research has shown that rural students’ online learning conditions, autonomous learning ability, and recognition are obviously weaker than those of urban students (Zhao et al., 2021). Therefore, ICTL may be more scarce for rural students (they less likely to have high ICTL), and improving rural students’ ICTL will have a stronger marginal effect on improving their SR compared with that of urban primary school students (Wu et al., 2019). Policymakers may need to pay more attention to improving rural students’ ICTL in the future to bridge the gap between urban and rural students in online learning (CNNIC, 2021).

## Limitations and future research

This study had the following limitations. First, the use of cross-sectional data made it difficult to deduce causality. Second, the OLSE of primary school students may be influenced by many other factors, such as the content and form of education, teaching attitudes, and teachers’ ICTL. Therefore, caution should be exercised when extending the research findings to explain online education in different countries and cultures. Third, the students sampled in this study were mainly from Guangdong province, whose economic and ICT development levels are higher than those of most other provinces in China (Sun et al., 2016). Therefore, the results may not explain the online education situation in other parts of China. Additional nationwide research is needed in China, especially in areas where levels of economic and ICTL development are lower than the national average, to better reveal the mechanism by which ICTL affects primary school students’ OLSE during the large-scale online education setting of the pandemic.

## Implications

To improve primary school students’ OLSE and SR, schools and education authorities need to strengthen the development of ICT-based education and the cultivation of students’ ICTL, such as by adding ICT-related courses, adjusting the curriculum content and teaching design according to online teaching requirements, evaluating the effectiveness of online learning in a timely manner, and strengthening cooperation between families and schools (Martin et al., 2021). The goal should be to improve the online learning ability of primary school students, especially those in rural areas. In the face of public health crises such as the COVID-19 pandemic, it is also necessary to make adequate plans and measures for the sudden demand of large-scale online education, strengthen technical support and psychological counseling for primary school students and their family members, pay attention to students’ learning experiences and emotional changes (Martin et al., 2021), and provide them with online and offline special guidance and remedial technical services (Yang, 2017). This may help them cope with challenges in the online education environment.

This study also revealed urban–rural differences in the relationship between ICTL and SR. In view of rural primary school students’ ICTL is significantly weaker than that of urban primary school students, and the greater marginal effect of improving the ICTL of rural (vs. urban) primary school students, more attention should be paid to this group when formulating relevant policies. The use of ICTs empowers different groups of learners to different degrees; that is, ICTs differ in their ability to help different groups of students solve learning problems. This is sometimes regarded as the new digital divide, compared with the first digital divide (differences in the possession of ICTDs) and the second (difference in ICTL) digital divide (Hohlfeld et al., 2008). Rural primary school students in China may need more ICT experience to empower them to deal with the difficulties and uncertainties of the online learning process (Martin et al., 2021). To solve the practical problems faced by rural primary school online learners in China, targeted online education methods and online curriculum models should be developed to suit rural learning environments (Zhao et al., 2021). For example, rural primary school students may lack effective supervision and guidance from their parents in the process of online learning, making them more likely to be distracted by online games and entertainment (Chang et al., 2016; Hansstein et al., 2017). To solve these problems, schools and education authorities should consider cooperating with technology enterprises to develop teaching aids and anti-Internet addiction technology (Martin et al., 2021) to improve the online learning of rural primary school students.

## Conclusion

Data on large-scale online education during the COVID-19 pandemic in China were used to assess the influence of primary school students’ ICTL on their OLSE, and the differences between rural and urban primary school students. The primary school students’ ICTL significantly predicted their OLSE, and SR played a mediating role. In addition, the RA/UA factor significantly moderated the first half of the mediation effect (ICTL to SR), indicating that the relationship between ICTL and SR was stronger among rural primary school students than among urban primary school students. These findings indicate that improving ICTL and SR is an effective way to improve primary school students’ OLSE. This should be considered in China’s online education policy, especially in underdeveloped areas (CNNIC, 2021). Compared with the urban sample, the sample of rural students was significantly weaker on ICTL, SR and OLSE, and showed a stronger positive correlation between ICTL and SR. This means that improving ICTL will show a stronger marginal positive effect on the SR of rural primary school students compared with urban primary school students.

## Data availability statement

The raw data supporting the conclusions of this article will be made available by the authors, without undue reservation.

## Ethics statement

The studies involving human participants were reviewed and approved by Guangzhou University. Written informed consent to participate in this study was provided by the participants' legal guardian/next of kin.

## Author contributions

JL and ML participated in study concepts, study design, literature research, data acquisition, and statistical analysis. XH, XL, and JW participated in study concepts, study design, and literature research. All authors contributed to the article and approved the submitted version.

## Funding

All sources of funding received for the research being submitted. This work was supported by grant from the National Educational Science Program of China: “Research on the Construction of Identity Education System for Hong Kong, Macao and Taiwan Students in Mainland Universities from the Perspective of Cultural Integration” (grant no. CIA200271).

## Conflict of interest

The authors declare that the research was conducted in the absence of any commercial or financial relationships that could be construed as a potential conflict of interest.

## Publisher’s note

All claims expressed in this article are solely those of the authors and do not necessarily represent those of their affiliated organizations, or those of the publisher, the editors and the reviewers. Any product that may be evaluated in this article, or claim that may be made by its manufacturer, is not guaranteed or endorsed by the publisher.
